# Neuroworsening in the Emergency Department Is a Predictor of Traumatic Brain Injury Intervention and Outcome: A TRACK-TBI Pilot Study

**DOI:** 10.3390/jcm12052024

**Published:** 2023-03-03

**Authors:** John K. Yue, Nishanth Krishnan, John H. Kanter, Hansen Deng, David O. Okonkwo, Ava M. Puccio, Debbie Y. Madhok, Patrick J. Belton, Britta E. Lindquist, Gabriela G. Satris, Young M. Lee, Gray Umbach, Ann-Christine Duhaime, Pratik Mukherjee, Esther L. Yuh, Alex B. Valadka, Anthony M. DiGiorgio, Phiroz E. Tarapore, Michael C. Huang, Geoffrey T. Manley, The TRACK-TBI Investigators

**Affiliations:** 1 Department of Neurological Surgery, University of California San Francisco, San Francisco, CA 94110, USA; 2 Brain and Spinal Injury Center, Zuckerberg San Francisco General Hospital, San Francisco, CA 94110, USA; 3 Section of Neurological Surgery, Dartmouth Hitchcock Medical Center, Lebanon, NH 03766, USA; 4 Department of Neurological Surgery, University of Pittsburgh Medical Center, Pittsburgh, PA 15261, USA; 5 Department of Emergency Medicine, University of California San Francisco, San Francisco, CA 94110, USA; 6 Department of Neurology, University of California San Francisco, San Francisco, CA 94110, USA; 7 Department of Neurological Surgery, Massachusetts General Hospital and Harvard Medical School, Boston, MA 02114, USA; 8 Department of Radiology and Biomedical Imaging, University of California San Francisco, San Francisco, CA 94110, USA; 9 Department of Neurological Surgery, University of Texas Southwestern Medical Center, Dallas, TX 75390, USA; 10 Institute for Health Policy Studies, University of California San Francisco, San Francisco, CA 94158, USA

**Keywords:** Glasgow Coma Scale, emergency department, mortality, neurological examination, neuroworsening, patient outcome assessment, traumatic brain injury

## Abstract

Introduction: Neuroworsening may be a sign of progressive brain injury and is a factor for treatment of traumatic brain injury (TBI) in intensive care settings. The implications of neuroworsening for clinical management and long-term sequelae of TBI in the emergency department (ED) require characterization. Methods: Adult TBI subjects from the prospective Transforming Research and Clinical Knowledge in Traumatic Brain Injury Pilot Study with ED admission and disposition Glasgow Coma Scale (GCS) scores were extracted. All patients received head computed tomography (CT) scan <24 h post-injury. Neuroworsening was defined as a decline in motor GCS at ED disposition (vs. ED admission). Clinical and CT characteristics, neurosurgical intervention, in-hospital mortality, and 3- and 6-month Glasgow Outcome Scale-Extended (GOS-E) scores were compared by neuroworsening status. Multivariable regressions were performed for neurosurgical intervention and unfavorable outcome (GOS-E ≤ 3). Multivariable odds ratios (mOR) with [95% confidence intervals] were reported. Results: In 481 subjects, 91.1% had ED admission GCS 13–15 and 3.3% had neuroworsening. All neuroworsening subjects were admitted to intensive care unit (vs. non-neuroworsening: 26.2%) and were CT-positive for structural injury (vs. 45.4%). Neuroworsening was associated with subdural (75.0%/22.2%), subarachnoid (81.3%/31.2%), and intraventricular hemorrhage (18.8%/2.2%), contusion (68.8%/20.4%), midline shift (50.0%/2.6%), cisternal compression (56.3%/5.6%), and cerebral edema (68.8%/12.3%; all *p* < 0.001). Neuroworsening subjects had higher likelihoods of cranial surgery (56.3%/3.5%), intracranial pressure (ICP) monitoring (62.5%/2.6%), in-hospital mortality (37.5%/0.6%), and unfavorable 3- and 6-month outcome (58.3%/4.9%; 53.8%/6.2%; all *p* < 0.001). On multivariable analysis, neuroworsening predicted surgery (mOR = 4.65 [1.02–21.19]), ICP monitoring (mOR = 15.48 [2.92–81.85], and unfavorable 3- and 6-month outcome (mOR = 5.36 [1.13–25.36]; mOR = 5.68 [1.18–27.35]). Conclusions: Neuroworsening in the ED is an early indicator of TBI severity, and a predictor of neurosurgical intervention and unfavorable outcome. Clinicians must be vigilant in detecting neuroworsening, as affected patients are at increased risk for poor outcomes and may benefit from immediate therapeutic interventions.

## 1. Introduction

Traumatic brain injury (TBI) is a prevalent cause of neurologic disability, comprising over 2 million emergency department (ED) visits, 275,000 hospitalizations, and 64,000 deaths in the United States (US) annually [1,2]. Initial management requires synthesis of clinical history, Glasgow Coma Scale (GCS) score, neurological examination, head computed tomography (CT) scan, and systemic injury evaluation [3,4,5]. An estimated 10% of moderate to severe TBI patients die within 6 months of their injury, and 15–20% are fully dependent for all activities of daily living [6]. While patients are generally not expected to die or incur severe disability after mild TBI (2% and 1–2% at 6 months post-injury, respectively), recent data suggest that 56% have not recovered to their functional baseline and 29% are unable to resume their prior level of employment [6,7]. Consequently, losses of livelihood and productivity for patients, their families, and society after TBI are immense [8,9,10].

Timely identification of patients at risk of neurologic deterioration can inform clinical triage to appropriate levels of care and treatment intervention. In 1998, Morris et al. defined neurologic deterioration (“neuroworsening”) as one or more of spontaneous decline in the GCS motor score by ≥2 points; new-onset loss of pupillary reactivity or development of pupillary asymmetry ≥2 mm; and deterioration in neurological or CT status sufficient to warrant immediate intervention [11]. Etiologies of neuroworsening are broad and encompass neurological, systemic, metabolic, and drug-induced causes. In the setting of acute TBI, culprit processes include expanding intracranial lesion(s), cerebral edema, and/or rising intracranial pressure (ICP) [12]. Reports have examined neuroworsening as either predictor or outcome in adult and pediatric TBI [13,14], e.g., to quantify the risk of hemorrhagic progression and in the context of antiplatelet or anticoagulant medications [15]. Additionally, the GCS motor score has been identified as a sensitive predictor of TBI outcomes and is widely used in prognostic calculators [8,9,10,16,17].

The definition of neuroworsening was revised by expert consensus to ≥1 point decrease in GCS motor score for assessment of severe TBI in the intensive care unit (ICU) at the 2019 Seattle International Severe Traumatic Brain Injury Consensus Conference (SIBICC) [18]. Herein, we evaluated the associations between neuroworsening in the ED (i.e., “early neuroworsening”) using the SIBICC definition, clinical severity and CT findings, neurosurgical intervention, in-hospital mortality, and 3- and 6-month outcomes in a prospectively enrolled cohort of acute TBI patients.

## 2. Materials and Methods

### 2.1. Study Overview

The Transforming Research and Clinical Knowledge in Traumatic Brain Injury Pilot (TRACK-TBI Pilot) study was a prospective, observational cohort study conducted at 3 US Level I trauma centers (Zuckerberg San Francisco General Hospital (San Francisco, California), University of Pittsburgh Medical Center (Pittsburgh, Pennsylvania), University Medical Center Brackenridge (Austin, Texas); ClinicalTrials.gov Registration: NCT01565551) using the National Institutes of Health (NIH) TBI Common Data Elements (CDEs) [19]. Inclusion criteria were external force trauma to the head, presentation to trauma center, and receiving a clinically indicated head CT scan <24 h after injury. Exclusion criteria were pregnancy, ongoing life-threatening disease (e.g., end-stage malignancy), police custody, involuntary psychiatric confinement, and inability to speak English (due to multiple outcome measures administered and/or normed only in English).

TRACK-TBI Pilot study procedures were conducted according to the ethical principles of the Declaration of Helsinki [20]. Eligible subjects were enrolled by convenience sampling during years 2010–2012, as previously described [19]. Institutional Review Board (IRB) approval was obtained at each participating center. The University of California, San Francisco Committee on Human Research provided overall study approval (protocol #10-00011). Informed consent was obtained from each subject or legally authorized representative prior to enrollment. Subjects enrolled by surrogate consent were re-consented by informed consent, if cognitively able, during the course of their clinical care or at follow-up time points for study participation.

**Figure 1:**
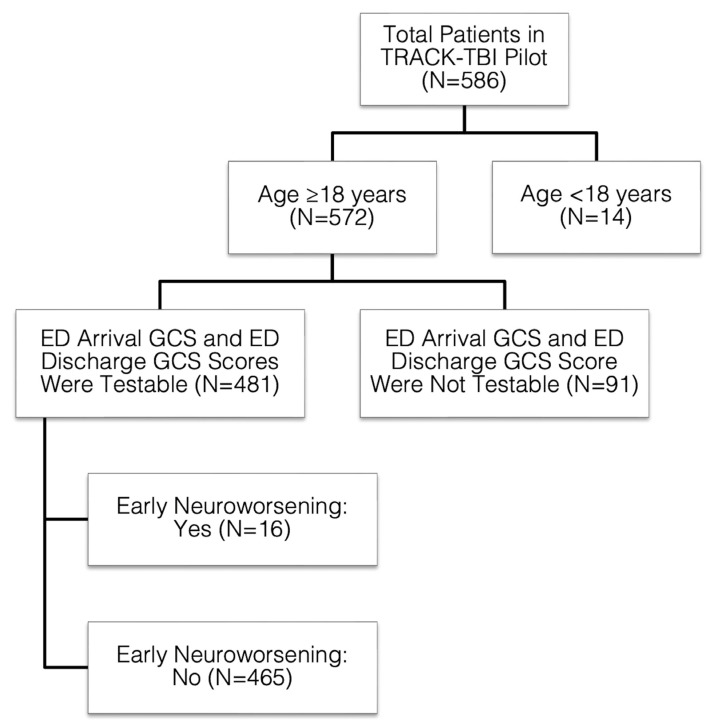
CONSORT Flow Diagram. Flowchart of included subjects. CONSORT = Consolidated Standards of Reporting Trials; ED = emergency department; GCS = Glasgow Coma Scale; TRACK-TBI Pilot = Transforming Research and Clinical Knowledge in Traumatic Brain Injury Pilot.

TRACK-TBI Pilot enrolled 586 subjects aged ≥16 years, as previously reported [18]. The current study is a secondary analysis of existing data. Data from subjects aged ≥18 years with testable values of the GCS eye, verbal, and motor scores upon ED arrival and at ED disposition were extracted from the TRACK-TBI Pilot database. Patients with GCS component scores coded as “untestable”, e.g., due to severe facial/eye swelling (eye score—untestable), intubation (verbal score—untestable), and deep sedation/paralysis (motor score—untestable), were excluded, yielding a final sample size of 481 for the current study (Figure 1). Early neuroworsening was defined as ≥1 point decrease in the GCS motor score between ED admission and ED disposition.

### 2.2. Demographic and Clinical Variables

Subjects were assessed by in-person interview and medical record review upon ED admission and enrollment. Subjects admitted to the hospital were followed until hospital discharge. Demographic, medical history, clinical, and injury history variables were collected in accordance with the NIH TBI CDEs, version 1 [21]. TBI severity was defined using the ED admission GCS score per current clinical standards (Severe TBI: GCS 3–8, Moderate: 9–12, Mild: 13–15) [22,23,24]. Data on time elapsed between ED admission and ED disposition were not available from TRACK-TBI Pilot.

### 2.3. Neuroimaging Variables and Coding

All subjects received a head CT within 24 h of injury as part of their clinical evaluation for TBI. Head CTs were read and coded by a central board-certified neuroradiologist blinded to subject characteristics, in accordance with the TBI CDEs for neuroimaging [25]. The Rotterdam CT score was selected to classify radiographic TBI severity, given its wide historical use across TBI studies and prognostic utility [26], and was coded by the same central blinded neuroradiologist as part of the TRACK-TBI Pilot [19].

### 2.4. Neurosurgical Intervention and In-Hospital Mortality

Cranial surgery, intracranial pressure (ICP) monitor placement, and in-hospital mortality were coded as yes/no in TRACK-TBI Pilot and were evaluated in the current study by early neuroworsening status. Timing of surgery, timing of ICP monitoring, type of surgery, and length of stay data were not available from TRACK-TBI Pilot.

### 2.5. 3- and 6-Month Outcomes

The Glasgow Outcome Scale-Extended (GOS-E) is an overall measure of functional disability based on consciousness, independence inside and outside the home, employability, social/community participation, and post-concussional symptomatology [27]. The 8-point ordinal scale consists of 1 = dead, 2 = vegetative state, 3 = lower severe disability (e.g., able to carry out activities of daily living (ADL) independently for less than 8 h per day), 4 = upper severe disability (e.g., able to carry out ADLs for more than 8 h per day), 5 = lower moderate disability (non-competitive work or inability to work, or inability to return to pre-injury social activities, or constant psychological disturbance), 6 = upper moderate disability (reduced work capacity, or >50% reduced social participation, or weekly psychological disturbance), 7 = lower good recovery (post-concussional symptoms, or <50% reduced social participation, or occasional psychological disruption), and 8 = upper good recovery (recovery to pre-injury status without new deficits). The GOS-E was administered through structured interviews by trained personnel via telephone at 3-months post-injury, and in-person at 6-months post-injury, as previously reported [19].

In TRACK-TBI Pilot, the GOS-E was administered to capture disability related to the TBI [28,29]. Based on data from recent TBI studies [6,30] and large neurosurgical clinical trials [31,32,33], GOS-E scores were dichotomized as unfavorable (GOS-E 1–3) vs. favorable (GOS-E 4–8). The rationale for including GOS-E scores of 4 as a favorable outcome is based on the premise that subjects able to function at home without supervision for greater than 8 h per day have considerable functional autonomy, and relatives overseeing their care can maintain full-time employment outside the home.

### 2.6. Statistical Analysis

Descriptive statistics were reported using means and standard deviations (SD) for continuous variables and proportions (%) for categorical variables. Early neuroworsening was the primary variable of interest and was coded as present/absent. Analysis of variance (ANOVA) and Pearson’s chi-squared test (χ2) were used to evaluate continuous and categorical dependent variables, respectively. Multivariable binary logistic regressions were performed for cranial surgery (dichotomized as yes/no), ICP monitoring (yes/no), and 3- and 6-month outcomes (GOS-E; dichotomized as unfavorable/favorable). Multivariable odds ratios (mOR) and their associated 95% confidence intervals [95% CI] were reported for predictors.

We recognized the risks of overfitting in our dataset, which had relatively small numbers of positive outcome events (cranial surgery: N = 25; ICP monitoring: N = 22; 3-month unfavorable outcome: N = 25; 6-month unfavorable outcome: N = 27), and thus did not seek to construct a multivariable model for in-hospital mortality (dead: N = 9). In consideration of the “rule of ten” for the number of dichotomous outcome events per predictor [34,35], we established a priori that our multivariable analyses would have at minimum 5 positive outcome events per predictor entered onto the model to mitigate the progressive risks of model overfitting. In addition to neuroworsening, our multivariable models for cranial surgery and ICP monitoring included age (≥65 vs. <65 years), ED admission GCS, and Rotterdam CT score, similar to prior analyses for surgery and prognostication after TBI [36,37,38]; for 3- and 6-month unfavorable outcome, polytrauma (extracranial Abbreviated Injury Scale score ≥3 in at least 1 body region; yes/no) [39] was added as a predictor. Age was dichotomized at 65 years, given evidence for its use in TBI and the geriatric literature as a threshold for increased baseline comorbidities and functional impairment that may precipitate TBI [40], increased likelihood of healthcare utilization after TBI [41,42], a high-risk factor for intracranial trauma and neurosurgical intervention per the Canadian Head CT Rule [43], and a standard cutoff for older age in validated TBI prognostic models [44] and assessment of TBI outcome [45].

Pupillary reactivity was initially considered as a secondary definition of neuroworsening and as a predictor for outcomes. However, it was not included due to the large degree of missing data in TRACK-TBI Pilot (pupillary reactivity was coded as “unknown/not done” in 47% of subjects at ED disposition).

Statistical significance was assessed at *p* < 0.05. Statistical analyses were performed using SPSS Statistics, version 29 (IBM Corporation, Chicago, IL, USA).

## 3. Results

### 3.1. Demographic and Presentation Characteristics

Overall, 481 adult subjects were extracted from TRACK-TBI Pilot, of which 16 (3.3%) had early neuroworsening. Detailed demographic, presentation, and injury characteristics are presented in Table 1. Overall, subjects had a mean age of 44.5 years (SD 18.0), 71.5% were male, and 79.8% were Caucasian. A greater proportion of neuroworsening subjects were aged ≥65 years (31.3% vs. 13.5%, *p* = 0.046; χ2), while no differences were observed for other demographic factors. A greater proportion of neuroworsening subjects had “unknown” post-traumatic amnesia (PTA; 75.0% vs. 11.8%, *p* < 0.001; χ2). Mechanism of injury, loss of consciousness, pupillary reactivity, antiplatelet and/or anticoagulation medication use, and potential confounders for depressed consciousness (urine drug screen, blood alcohol screen, and polytrauma) did not differ by neuroworsening status.

Overall, the majority of subjects were classified as mild TBI (ED admission GCS 13–15: 91.1%). Mean ED admission GCS was 14.0 (SD 2.6) and was significantly lower in neuroworsening subjects (9.9 (SD 3.3) vs. 14.1 (SD 2.5), *p* < 0.001; ANOVA). There were higher proportions of moderate and severe TBI in neuroworsening subjects (GCS 9–12: 31.3% vs. 2.6%; GCS 3–8: 37.5% vs. 4.3%, respectively, *p* < 0.001; χ2). Notably, 31.3% of neuroworsening subjects had ED admission GCS 13–15. ED disposition was significantly lower in neuroworsening subjects (3.3 (SD 1.0) vs. 14.4 (SD 2.1), *p* < 0.001; ANOVA). Notably, all neuroworsening subjects declined to GCS 3–8 at ED disposition and required ICU admission (vs. 26.2% of non-neuroworsening with ICU admission; *p* < 0.001; χ2). Neuroworsening was associated with intravenous hyperosmolar infusion in the ED (mannitol or 23.4% hypertonic saline; 12.5% vs. 6.2%, *p* < 0.001; χ2).

### 3.2. Radiographic Intracranial Injury Characteristics

Detailed CT characteristics are presented in Table 2. Overall, 47.2% were positive for acute intracranial injury on CT consistent with TBI. Notably, 100% of subjects with early neuroworsening were CT-positive (vs. 45.4%, *p* < 0.001; χ2). Early neuroworsening was associated with significantly higher rates of the majority of lesion types, including subdural hematoma (SDH; 75.0% vs. 22.2%, *p* < 0.001; χ2), subarachnoid hemorrhage (SAH; 81.3% vs. 31.2%, *p* < 0.001; χ2), contusion (68.8% vs. 20.4%, *p* < 0.001; χ2), and intraventricular hemorrhage (IVH; 18.8% vs. 2.2%, *p* < 0.001; χ2). Epidural hematoma (EDH) and diffuse axonal injury (DAI) did not differ by neuroworsening status. Early neuroworsening was also associated with significantly elevated rates of signs of herniation: midline shift (50.0% vs. 2.6%, *p* < 0.001; χ2), cisternal compression (56.3% vs. 5.6%, *p* < 0.001; χ2), and cerebral edema (68.8% vs. 12.3%, *p* < 0.001; χ2). Rotterdam CT scores were higher in subjects with early neuroworsening (1 through 6: 0.0%, 12.5%, 31.3%, 18.8%, 25.0%, 12.5% vs. 1.1%, 71.2%, 23.7%, 2.6%, 1.3%, 0.2%, respectively, *p* < 0.001; χ2).

### 3.3. Neurosurgical Intervention and In-Hospital Mortality

**Figure 2:**
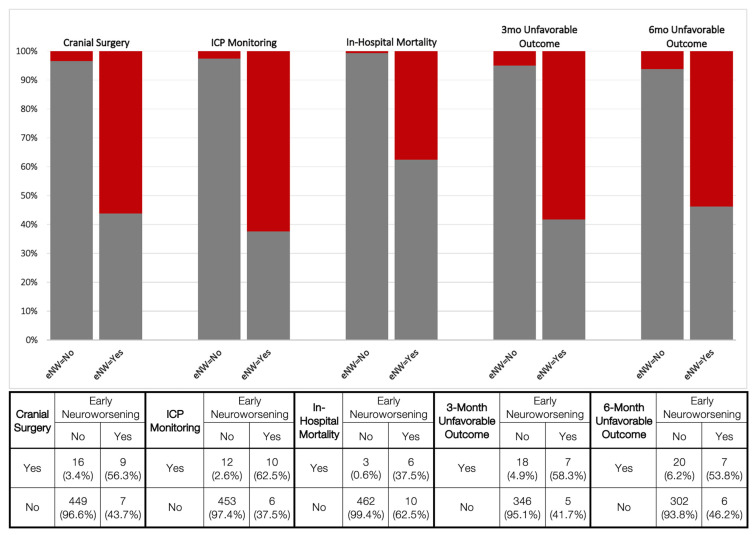
Interventions and outcomes by early neuroworsening. Proportions of presence (red) or absence (gray) of neurosurgical intervention (cranial surgery; ICP monitoring), in-hospital mortality, and 3- and 6-month unfavorable outcome (Glasgow Outcome Scale-Extended score of 1–3) are displayed by early neuroworsening (eNW) status. Column percentages are provided in the table. All comparisons were statistically significant with *p* < 0.001. eNW = early neuroworsening; ICP = intracranial pressure; mo = month.

Acute neurosurgical interventions consisted of cranial surgery and/or insertion of an ICP monitor, and rates for both were considerably higher in early neuroworsening subjects (cranial surgery: 56.3% vs. 3.4%, *p* < 0.001; χ2; ICP monitor: 62.5% vs. 2.6%, *p* < 0.001; χ2). In-hospital mortality was considerably higher in neuroworsening subjects (37.5% vs. 0.6%; *p* < 0.001; χ2) (Figure 2). On multivariable logistic regressions, these associations were conserved; neuroworsening remained a predictor for undergoing cranial surgery (mOR 4.65, 95% CI [1.02–21.19]; *p* = 0.047) and ICP monitoring (mOR 15.48, [2.92–81.85]; *p* = 0.001) (Table 3).

### 3.4. 3- and 6-Month Outcomes

The incidences of 3- and 6-month unfavorable outcomes were higher in neuroworsening subjects (58.3% vs. 4.9%, p < 0.001; 53.8% vs. 6.2%, *p* < 0.001, respectively; χ2) (Figure 2). These associations were conserved on multivariable logistic regressions for unfavorable outcome at 3 months (neuroworsening: mOR 5.36 [1.13–25.36]; *p* = 0.034) and 6 months post-injury (mOR 5.68 [1.18–27.35]; *p* = 0.030) (Table 3).

## 4. Discussion

Neuroworsening is a sign of progressive neurologic injury following TBI. We defined early neuroworsening as a ≥1 point decrease in GCS motor score between ED admission and ED disposition. In the present prospective cohort of 481 acute TBI subjects, most of whom had GCS 13–15 on ED admission, neuroworsening subjects were considerably more likely to have elevated lesion burden on CT, neurosurgical intervention, in-hospital mortality, and unfavorable outcome. On multivariable analysis, early neuroworsening predicted the need for cranial surgery, ICP monitoring, and 3- and 6-month unfavorable outcome. These data show that neuroworsening during the ED phase of care is an indicator of TBI severity, imminent decline, and need for care escalation.

### 4.1. Neuroworsening Is an Early Indicator of Brain Injury Severity

Our study showed that neuroworsening can be used as an indicator of evolving brain injury in the ED evaluation of TBI. Neuroworsening subjects had lower mean ED admission GCS score by 4 points (10 vs. 14), 100% CT-positivity, and increased CT lesion burden. The incidence of IVH was 9-fold higher in neuroworsening subjects (19% vs. 2%), which has been shown to confer 3.8-fold odds of in-hospital mortality [46]. In our study, the high rates of SDH, SAH, and contusion in neuroworsening subjects (68–81% vs. 20–31%), combined with CT signs of cerebral edema (69% vs. 12%) and herniation (50–56% vs. 3–6%), describe more severe primary injuries with evolving secondary injuries that portend poorer prognoses across the spectrum of TBI. Indeed, a large multicenter, externally validated study of 1935 mild TBI subjects showed that SDH, SAH, and/or contusions on initial head CT correlated with 12-month incomplete recovery (odds ratio 1.8–2.7) and unfavorable outcome (odds ratio 1.7–3.2), and IVH had an odds ratio of 3.5 for 12-month unfavorable outcome [47].

Concordantly, neuroworsening was associated with higher Rotterdam CT scores. A total of 56% of neuroworsening subjects (vs. 4%) had a Rotterdam score ≥4, which is associated with 8- to 11-fold odds of in-hospital mortality [48,49]. For reference, Rotterdam scores of 5 and 6 confer 53% and 61% risk of 6-month mortality, respectively [26].

DAI and EDH were the only CT lesion types that did not statistically differ by neuroworsening status. DAI is not a lesion type associated with focal mass effect and hence is less likely to cause fluctuations in the GCS motor exam during emergency care. Classically, EDHs may present with a “lucid interval” where the patient wakes from initial unconsciousness prior to secondary deterioration, 12–42% remain conscious from time of injury to time of cranial surgery, and up to 27% remain neurologically intact [50]. EDH was the second least frequent lesion type (4%) in our study above IVH (2.7%). While reasons for the lack of observed association in our data are unclear, small sample size, higher likelihood of associated wakefulness, venous etiologies with slower progression, and time elapsed in the ED prior to hospital admission are all possible explanations and warrant further analysis in studies where follow-up CTs are available.

Neuroworsening subjects were older, with a larger proportion aged ≥65 years (31 vs. 14%). Studies have shown that compared to younger patients, those aged ≥65 years who seek emergency room care for TBI are 3 times more likely to receive a head CT or MRI, 4 times more likely to be admitted to the hospital [51], and 2–4 times more likely to have imaging evidence of acute intracranial trauma after mild TBI [52]. Older age has been associated with anatomical changes, e.g., cerebral atrophy and larger extra-axial spaces [53], and well as physiologic changes, e.g., increased comorbidities/frailty [54,55] and increased prevalence of antiplatelet and anticoagulant medications [56,57], all of which may contribute to altered risks of neurologic deterioration and/or hemorrhagic progression [57,58]. These factors warrant further study in larger datasets.

### 4.2. Neuroworsening Is a Predictor of Neurosurgical Interventions, Mortality, and Outcome

All subjects with neuroworsening in the ED were admitted to ICU, and neuroworsening predicted the need for cranial surgery and ICP monitoring with multivariable odds ratios of 5 and 15, respectively. This is not surprising, as neuroworsening was associated with markedly increased CT lesion burden and severity. Consequently, the mortality prevalence was 60-fold higher for neuroworsening (44% vs. <1%). Subjects with neuroworsening were more likely to have 3- and 6-month unfavorable outcome (58% vs. 5%; 54% vs. 6%) with multivariable odds ratios of 5.4–5.7, underscoring the importance of neuroworsening in the ED as an independent predictor of poor functional outcome.

Consensus guidelines in severe TBI have established the importance of evaluating critical neuroworsening for the neurologically debilitated ICU patient [12]. While ICP and multimodal intracranial monitoring are valuable tools for detecting clinical deterioration, the benchmark remains serial clinical examinations to guide triage decisions for CT neuroimaging [59]. Our findings extend these recommendations to ED and acute care settings, where the relevance of early detection of neuroworsening can be applied to non-neurologically devastated patients who are at high risk for deterioration.

Of note, the proportion of subjects with early neuroworsening in our study was limited. Contributing factors include applying the rigorous definition of neuroworsening consistent with SIBICC (≥1 point decline on the GCS motor score) [18], within the ED phase of care, in a predominantly mild TBI cohort. To date, studies on neuroworsening have targeted cohorts within TBI severity strata (mild/moderate/severe) or by acute CT findings. A 2018 meta-analysis of 49 mild TBI studies found a pooled prevalence of 11.7% for clinical deterioration, 3.5% for neurosurgical intervention, and 1.4% for mortality [60]. Deterioration was most often defined as progression of brain injury on CT scan, and supplemented by the clinical exam when available [60], across a variable timeframe ranging from 24 to 72 h of arrival to anytime during acute hospitalization [13,61,62,63]. These observations were corroborated by smaller, single-institution studies, e.g., a Polish study of 186 hospitalized mild TBI patients defined neuroworsening as ≥1 point decline on the GCS *total* score at any point during hospitalization, and reported neuroworsening in 3.8% and neurosurgical intervention in 1.6% [64]. Overall, our results were comparable to prior studies (neuroworsening: 3.3%, cranial surgery: 5.2%, in-hospital mortality: 1.9%, 6-month unfavorable outcome: 8.1%) [60,64,65], and importantly showed profound differences in intracranial injury severity, therapeutic intensity, mortality, and unfavorable outcome associated with neuroworsening identified within the early phase of care.

### 4.3. Implications for ED and Acute Care Clinicians

The high prevalence of multifocal TBI and the worsened prognosis based on severity of CT features highlight neuroworsening as a sign that should be recognized without delay in ED and acute care settings, similar to the established guidelines for critical neuroworsening in the ICU [18]. Our study shows that subjects with early neuroworsening have elevated risks of long-term morbidity and mortality, and a greater likelihood of requiring intensive care and interventions including ICU admission, intracranial neuromonitoring, and surgery. Given the risks of neurologic deterioration even in mild TBI [66,67], any TBI patient in the ED with suspected neurologic deficit or positive head CT should continue to undergo short-interval GCS assessments by trained clinicians beyond the time of ED admission, including when awaiting clinical decision making or hospital admission, as significant time may elapse before a patient completes ED disposition. The first sign of neuroworsening should trigger immediate, formal neurologic evaluation. The optimal interval for GCS reassessments constitutes an important area for future study.

The GCS motor score is a known predictor of mortality and unfavorable outcome [33,68,69] and has been incorporated into validated TBI prognostic calculators [70]. The neurotrauma clinician must understand and accurately apply the GCS motor score as part of TBI standard of care. The GCS motor score is often considered the most difficult of the three GCS components to administer, even in experienced healthcare providers [71]. Training and education have been shown to substantially improve assessment accuracy: in a 2016 study of 54 surgical trauma, neurotrauma, and neurovascular ICU nurses at a US Level I trauma center, the accuracy of GCS motor score assessments improved from 50% to 93% after participants completed a mandatory neuroscience course [71]. Our findings support the rationale for providing formal training to providers in ED settings regarding not only the correct use of the GCS motor score, but also how to recognize a clinical change in the motor score and its implications for escalation of care.

### 4.4. Limitations

We acknowledge important limitations to the interpretation of our data. While our overall sample size of 481 was reasonable, only 3.3% had early neuroworsening, which limited between-group comparisons due to the inherent heterogeneity of presentation and injury within small samples. To maximize our group sizes for meaningful statistical comparisons, we combined neuroworsening subjects into a single group and did not control for the magnitude of scalar decrease in the GCS motor score from ED admission to disposition. Whether the magnitude of neuroworsening, or the rate of change, predicts intervention or outcomes requires further study. We aimed to balance the risk of model overfitting with the small proportion of neuroworsening subjects by setting an *a priori* threshold of five positive outcome events per entered predictor in multivariable logistic regressions; however, the residual risk of overfitting is present, as are potential contributions from factors not controlled for in our regressions (e.g., frailty), which limit generalizability. We were unable to include other known criteria for ICU neuroworsening [18], such as pupillary reactivity (due to high degree of data missingness) and serial CT data (not available in TRACK-TBI Pilot), which further limits data interpretation. As pupillary reactivity data from TRACK-TBI Pilot were extracted from the medical record, the degree of missingness should serve as a reminder to clinicians regarding the importance of systematically documenting pupillary reactivity throughout the ED clinical course, including ED admission and disposition. While our approach to outcome dichotomization conformed to prior published analyses [6,30], we recognize that dichotomization, in general, confers known disadvantages such as underestimating the variance in outcome between the dichotomized groups, reducing statistical power, and detecting a falsely positive result [72,73]. For these reasons, our findings should be interpreted cautiously, and confirmation in larger datasets and larger cohorts of subjects with early neuroworsening is needed, which will improve the rigor of statistical inference and accuracy of multivariable models. Future investigations on neurologic deterioration that incorporate serial imaging data for analyses of lesion progression should consider the evaluation of home antiplatelet and anticoagulant medications (and their indications), standard hematology and coagulation laboratory values, and whether blood products or coagulopathy reversal agents were administered.

Data on timing (e.g., of ED disposition GCS score, cranial surgery, ICP monitoring placement, and hospital discharge) were not available from TRACK-TBI Pilot, which precluded time course analyses on medical decision making (such as time to surgical intervention) and the relationships among time to presentation, time to neuroworsening, and time to initial CT. For example, it is possible that pre-hospital transport time influences the relative time it takes an at-risk subject to deteriorate, or that their neuroworsening was expected given the known severity and specific pathoanatomic character of their TBI. Our aim was to evaluate the clinical factors and risks associated with “early” neuroworsening in the ED and did not include analyses for subjects with neuroworsening further into their hospital course, which constitutes a separate cohort. A 2022 retrospective study of 458 TBI subjects found a mean difference of 1.1 points between GCS scores recorded by the trauma registry and neurosurgery consultation [74], highlighting the importance of capturing timing of GCS assessment in reference to time of injury, ED admission, and interventions, in order to better determine the prognostic value of early neuroworsening and optimal timing of reassessments.

Prognostication of TBI outcome is multifactorial and requires careful consideration of potential confounders. Detailed hospital intervention data, including types of cranial and extracranial surgeries, medical interventions, and complications, were not available in our dataset, which limited our interpretation of causality in hospital and long-term outcomes. For the latter, rehabilitation and post-acute care information were also not available. While we included only subjects with testable ED GCS as defined by the TBI CDEs (e.g., without deep sedation or paralysis to confound the motor score), ED medications were not recorded in the TRACK-TBI Pilot study, and unrecorded sedatives or paralytics could be confounders of ED disposition GCS scores. While we controlled for age in our analyses, the impact of older age on neuroworsening in the ED and the resultant clinical course is an important topic for future study. These limitations require investigation in cohorts of sufficient size and data granularity, which we plan to undertake using the recently completed, 18-center Transforming Research and Clinical Knowledge in Traumatic Brain Injury study (ClinicalTrials.gov Registration: NCT02119182).

## 5. Conclusions

Neuroworsening in the ED following traumatic brain injury is an early indicator of clinical and radiographic TBI severity and is an independent predictor of neurosurgical intervention and 3- and 6-month unfavorable outcome when controlling for ED admission GCS. Clinicians in ED and acute care settings caring for TBI patients should be vigilant in detecting neuroworsening, as affected patients are at increased risk for poor outcomes and may benefit from immediate therapeutic interventions.

## Figures and Tables

**Table 1:** Demographic and presentation characteristics by early neuroworsening. Demographic, clinical, and injury variables compared by early neuroworsening status. Only a subset of subjects had recorded data for ED blood alcohol screen (group sizes (N) are shown). ED hyperosmolar therapy included administration of intravenous mannitol or 23.4% hypertonic saline. ED = emergency department; GCS = Glasgow Coma Scale; SD = standard deviation; TBI = traumatic brain injury.

Variable	Overall (N = 481)	Early Neuroworsening: Yes (N = 16)	Early Neuroworsening: No (N = 465)	Sig. (*p*)
Age (Years)				
Mean (SD)	44.5 (18.0)	53.4 (20.5)	44.2 (17.9)	0.044
≥65 Years	68 (14.1%)	5 (31.3%)	63 (13.5%)	0.046
Male	344 (71.5%)	12 (75.0%)	332 (71.4%)	0.754
Race				0.305
Caucasian/White	384 (79.8%)	15 (93.8%)	369 (79.4%)	
African-American/African	44 (9.1%)	1 (6.3%)	43 (9.2%)	
Other Races	53 (11.0%)	0 (0.0%)	53 (11.0%)	
Education (Years)				0.313
Mean (SD)	13.8 (3.0)	14.7 (3.8)	13.8 (2.9)	
Current Antiplatelet and/or Anticoagulant Medication	69 (14.3%)	4 (25.0%)	65 (14.0%)	0.216
ED Admission GCS Score				
Mean (SD)	14.0 (2.6)	9.9 (3.3)	14.1 (2.5)	<0.001
3–8	26 (5.4%)	6 (37.5%)	20 (4.3%)	<0.001
9–12	17 (3.5%)	5 (31.3%)	12 (2.6%)	
13–15	438 (91.1%)	5 (31.3%)	433 (93.1%)	
ED Disposition GCS Score				
Mean (SD)	14.0 (2.9)	3.3 (1.0)	14.4 (2.1)	<0.001
3–8	34 (7.1%)	16 (100.0%)	18 (3.9%)	
9–12	7 (1.5%)	0 (0.0%)	7 (1.5%)	
13–15	440 (91.5%)	0 (0.0%)	440 (94.6%)	
Mechanism of Injury				0.863
Motor Vehicle Accident	78 (16.3%)	2 (12.5%)	76 (16.4%)	
Motorcycle Crash	26 (5.4%)	1 (6.3%)	25 (5.4%)	
Pedestrian Struck by Vehicle	56 (11.7%)	2 (12.5%)	54 (11.6%)	
Fall From Moving Object	60 (12.5%)	2 (12.5%)	58 (12.5%)	
Fall From Standing/Stationary Object	161 (33.5%)	8 (50.0%)	153 (33.0%)	
Assault	82 (17.1%)	1 (6.3%)	81 (17.5%)	
Other Mechanism	18 (3.5%)	0 (0.0%)	3 (0.6%)	
Loss of Consciousness				0.600
No	116 (24.1%)	2 (12.5%)	114 (24.5%)	
Yes	331 (68.8%)	12 (75.0%)	319 (68.6%)	
Unknown	34 (7.1%)	2 (12.5%)	32 (6.9%)	
Post-Traumatic Amnesia				<0.001
No	155 (32.2%)	0 (0.0%)	155 (33.3%)	
Yes	259 (53.8%)	4 (26.7%)	255 (54.8%)	
Unknown	67 (14.0%)	12 (75.0%)	55 (11.8%)	
ED Admission Pupillary Reactivity				0.999
Both Reactive	405 (84.2%)	14 (87.5%)	391 (84.0%)	
One Non-Reactive	6 (1.2%)	0 (0.0%)	6 (1.3%)	
Both Non-Reactive	7 (1.5%)	0 (0.0%)	7 (1.5%)	
Unknown/Not Done	63 (13.1%)	2 (12.5%)	61 (13.1%)	
ED Disposition Pupillary Reactivity				0.338
Both Reactive	252 (52.3%)	11 (68.8%)	241 (51.8%)	
One Non-Reactive	0 (0.0%)	0 (0.0%)	0 (0.0%)	
Both Non-Reactive	6 (1.2%)	0 (0.0%)	5 (1.1%)	
Unknown/Not Done	224 (46.5%)	5 (31.3%)	219 (47.0%)	
Polytrauma	76 (15.8%)	2 (12.5%)	74 (15.9%)	0.713
ED Urine Drug Screen Positive	31 (6.4%)	2 (12.5%)	29 (6.2%)	0.316
ED Blood Alcohol Screen Positive	N = 229	N = 12	N = 217	0.516
No	132 (57.6%)	8 (66.7%)	124 (57.1%)	
Yes	97 (42.4%)	4 (33.3%)	93 (42.9%)	
ED Hyperosmolar Therapy	8 (1.7%)	2 (12.5%)	6 (1.3%)	<0.001
ED Disposition				<0.001
Home	149 (31.0%)	0 (0.0%)	149 (32.0%)	
Ward	194 (40.3%)	0 (0.0%)	194 (41.7%)	
Intensive Care Unit	138 (28.7%)	16 (100.0%)	122 (26.2%)	

**Table 2:** Initial head CT characteristics by early neuroworsening. Presence of intracranial injury on initial head CT scan, and major types of intracranial pathology, are shown by early neuroworsening status. The Rotterdam CT score provides the following estimates for 6-month mortality after traumatic brain injury: 1 = 0%, 2 = 7%, 3 = 16%, 4 = 26%, 5 = 53%, 6 = 61%. CT = computed tomography; SD = standard deviation.

Variable	Overall (N = 481)	Early Neuroworsening: Yes (N = 16)	Early Neuroworsening: No (N = 465)	Sig. (*p*)
CT Intracranial Injury Present	227 (47.2%)	16 (100%)	211 (45.4%)	<0.001
Epidural Hematoma	19 (4.0%)	0 (0.0%)	19 (4.1%)	0.409
Subdural Hematoma	115 (23.9%)	12 (75.0%)	103 (22.2%)	<0.001
Subarachnoid Hemorrhage	158 (32.8%)	13 (81.3%)	145 (31.2%)	<0.001
Contusion	106 (22.0%)	11 (68.8%)	95 (20.4%)	<0.001
Intraventricular Hemorrhage	13 (2.7%)	3 (18.8%)	10 (2.2%)	<0.001
Diffuse Axonal Injury	34 (7.1%)	2 (12.5%)	32 (6.9%)	0.389
Midline Shift	20 (4.2%)	8 (50.0%)	12 (2.6%)	<0.001
Cisternal Compression	35 (7.3%)	9 (56.3%)	26 (5.6%)	<0.001
Cerebral Edema	68 (14.1%)	11 (68.8%)	57 (12.3%)	<0.001
Rotterdam CT Score				
Mean (SD)	2.4 (0.7)	3.9 (1.3)	2.3 (0.6)	<0.001
=1	5 (1.0%)	0 (0.0%)	5 (1.1%)	<0.001
=2	333 (69.2%)	2 (12.5%)	331 (71.2%)	
=3	115 (23.9%)	5 (31.3%)	110 (23.7%)	
=4	15 (3.1%)	3 (18.8%)	12 (2.6%)	
=5	10 (2.1%)	4 (25.0%)	6 (1.3%)	
=6	3 (0.6%)	2 (12.5%)	1 (0.2%)	

**Table 3:** Multivariable regression for interventions and outcomes. Multivariable binary logistic regressions were performed for four dependent measures: cranial surgery, ICP monitoring, 3-month unfavorable outcome, and 6-month unfavorable outcome. Unfavorable outcome was defined as Glasgow Outcome Scale-Extended score of 1–3. Early neuroworsening was the variable of interest. Multivariable odds ratios (mOR) and their associated [95% confidence intervals (CI)] are shown for each per-unit increase of the predictor variable. CT = computed tomography; ED = emergency department; GCS = Glasgow Coma Scale; ICP = intracranial pressure.

Cranial Surgery		
Predictor	mOR [95% CI]	Sig. (*p*)
Early Neuroworsening	4.65 [1.02–21.19]	0.047
Age ≥ 65 Years	0.96 [0.17–5.40]	0.965
ED Admission GCS	0.76 [0.68–0.86]	<0.001
Rotterdam CT Score	4.12 [2.13–7.97]	<0.001
**ICP Monitoring**		
Predictor	mOR [95% CI]	Sig. (*p*)
Early Neuroworsening	15.48 [2.92–81.85]	0.001
Age ≥ 65 Years	1.05 [0.17–6.37]	0.959
ED Admission GCS	0.65 [0.55–0.76]	<0.001
Rotterdam CT Score	1.93 [1.07–3.48]	0.03
**3-Month Unfavorable Outcome**		
Predictor	mOR [95% CI]	Sig. (*p*)
Early Neuroworsening	5.36 [1.13–25.36]	0.034
Age ≥ 65 Years	4.61 [1.56–13.58]	0.006
ED Admission GCS	0.80 [0.71–0.91]	<0.001
Rotterdam CT Score	1.73 [0.95–3.16]	0.073
Polytrauma	1.81 [0.60–5.52]	0.295
**6-Month Unfavorable Outcome**		
Predictor	mOR [95% CI]	Sig. (*p*)
Early Neuroworsening	5.68 [1.18–27.35]	0.030
Age ≥ 65 Years	7.22 [2.53–20.55]	<0.001
ED Admission GCS	0.76 [0.67–0.86]	<0.001
Rotterdam CT Score	0.97 [0.53–1.78]	0.928
Polytrauma	1.52 [0.52–4.51]	0.446

## Data Availability

The data presented herein are available in the Federal Interagency Traumatic Brain Injury Research (FITBIR) Informatics System at doi:10.23718/FITBIR/1419836, and upon request to the corresponding author. Qualified researchers can request access to data stored in FITBIR. To gain access to shared data, an investigator must obtain data access privileges.
